# Medication Exposure and Mortality in Patients With Schizophrenia

**DOI:** 10.1001/jamanetworkopen.2024.47137

**Published:** 2024-11-22

**Authors:** Sébastien Brodeur, Yohann M. Chiu, Josiane Courteau, Marc Dorais, Dominic Oliver, Emmanuel Stip, Marie-Josée Fleury, Marc-André Roy, Alain Vanasse, Alain Lesage, Jacinthe Leclerc

**Affiliations:** 1 Département de Psychiatrie et Neurosciences, Université Laval, Québec City, Québec, Canada; 2 Centre de Recherche CERVO, Québec City, Québec, Canada; 3 Département de Médecine de Famille et de Médecine D'urgence, Université de Sherbrooke, Sherbrooke, Québec, Canada; 4 Groupe de Recherche PRIMUS, Centre de Recherche du Centre Hospitalier Universitaire de Sherbrooke (CRCHUS), Sherbrooke, Québec, Canada; 5 StatSciences Inc, Notre-Dame-de-Île-Perrot, Québec, Canada; 6 Department of Psychiatry, University of Oxford, London, United Kingdom; 7 Département de Psychiatrie et d'Addictologie, Université de Montréal, Montréal, Québec, Canada; 8 Department of Psychiatry, McGill University, Montréal, Québec, Canada; 9 Douglas Mental Health University Institute, McGill University, Montréal, Québec, Canada; 10 Centre de Recherche, Institut Universitaire en Santé Mentale de Montréal, Montréal, Québec, Canada; 11 Faculté de Pharmacie, Université Laval, Québec City, Québec, Canada; 12 Centre de Recherche de l'Institut Universitaire de Cardiologie et de Pneumologie de Québec-Université Laval, Québec City, Québec, Canada

## Abstract

**Importance:**

The use of antipsychotics, antidepressants, and benzodiazepines may influence the risk of mortality in people with schizophrenia. However, many observational studies have not accounted for immortal time bias (ITB), which occurs when there is a period during which patients in the exposed group are necessarily alive and misclassified as exposed (the period between start of follow-up and initiation of drug). Ignoring ITB may lead to misinterpretation of the association between these drugs and mortality.

**Objectives:**

To examine whether the cumulative dose of antipsychotics, antidepressants, and benzodiazepines is associated with mortality risk in patients with schizophrenia and discuss the potential impacts of ignoring ITB.

**Design, Setting, and Participants:**

This cohort study used administrative data from Québec, Canada, including patients aged 17 to 64 years diagnosed with schizophrenia between January 1, 2002, and December 31, 2012. Data analysis was performed from June 22, 2022, to September 30, 2024.

**Main Outcomes and Measures:**

The primary outcome was all-cause mortality, with follow-up from January 1, 2013, to December 31, 2017, or until death. Mortality risk was assessed for low, moderate, and high exposure to antipsychotics, antidepressants, and benzodiazepines. Cox proportional hazards regression models with time-fixed exposure (not controlling for ITB) and time-dependent exposure (controlling for ITB) were performed.

**Results:**

The cohort included 32 240 patients (mean [SD] age, 46.1 [11.6] years; 19 776 [61.3%] men), of whom 1941 (6.0%) died during follow-up. No dose-response association was found for antipsychotics with mortality using the time-fixed method. However, high-dose antipsychotic use was associated with increased mortality after correcting for ITB (adjusted hazard ratio [AHR], 1.28; 95% CI, 1.07-1.55; *P* = .008). Antidepressants showed a reduced mortality risk using the time-fixed method, but only at high doses when correcting for ITB (AHR, 0.86; 95% CI, 0.74-1.00; *P* = .047). Benzodiazepines were associated with increased mortality risk regardless of the method.

**Conclusions and Relevance:**

The findings of this study do not dispute the known efficacy of antipsychotics in schizophrenia, but they call into question the magnitude of long-term mortality benefits.

## Key Points

**Question:**

What is the role of not controlling for immortal time bias (ITB) in studies evaluating the association between antipsychotics, antidepressants, and benzodiazepines and all-cause mortality in patients with schizophrenia?

**Findings:**

In a cohort study of 32 240 patients with schizophrenia, not controlling for ITB overestimated the protective association of antipsychotics and antidepressants and underestimated the harmful association of benzodiazepine use at lower doses. After controlling for ITB, high-dose antipsychotic and benzodiazepine use was associated with increased mortality and the protective association of antidepressants was not found.

**Meaning:**

The results of this study do not invalidate the known effectiveness of antipsychotics in people diagnosed with schizophrenia, but question the extent of their protective association on long-term mortality.

## Teaser

This cohort study examines the association of antipsychotic, antidepressant, and benzodiazepine therapy with all-cause mortality in patients with schizophrenia and assesses the role of controlling for immortal time bias in determining this association.

## Introduction

Schizophrenia is a serious psychiatric disorder affecting 287 individuals per 100 000 worldwide.^1^ People with schizophrenia have a life expectancy 15 years shorter than the general population and a mortality rate almost 3 times higher.^2^

Antipsychotic treatment is crucial for managing the symptoms of schizophrenia but it can also be associated with adverse effects, including cardiometabolic risks.^3,4^ Despite these effects, antipsychotics have shown a moderate decreased risk of all-cause mortality, including suicide and cardiovascular issues,^5,6^ as replicated across various studies,^2,7^ indicating improved survival at different antipsychotic dosages^8^ and age groups.^9^ Adjunctive psychotropic treatments for common psychiatric comorbidities also influence survival rates. For instance, antidepressant use in patients with schizophrenia has been associated with a reduced mortality risk,^10^ with varying associations depending on the level of exposure.^7^ In Taiwan, exposure to low and moderate doses of antidepressants reduced all-cause mortality.^8^ Conversely, benzodiazepines and sedative-hypnotics were associated with an increased risk of death in schizophrenia.^7,8,9^

**Figure 1.**
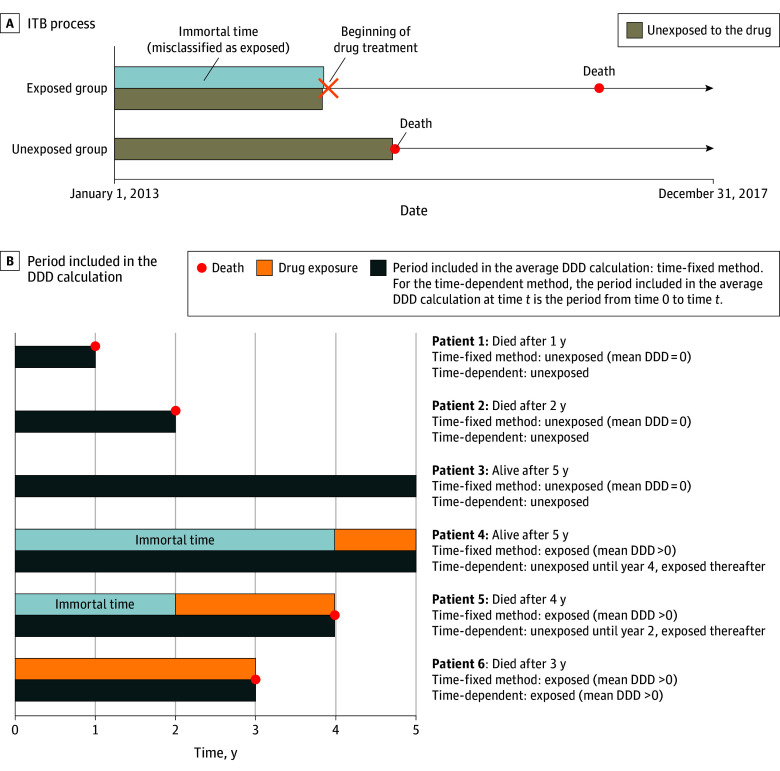
Illustration of the Immortal Time Bias (ITB) in the Context of Drug Exposure and Mortality Period during which the outcome of the exposure cannot occur (A), and time-dependent method of analysis (B). The ITB is a type of bias occurring when, among the exposed group, there is a follow-up period between study entry and the determination of the exposure variable in which the outcome cannot occur, resulting in an artificially lower incidence of the event in the exposed group compared with the unexposed group.^11^ Another way of seeing the ITB is the following: ITB occurs when, during follow-up, cohort members develop the outcome of interest before they had the chance to be exposed to the drug because the follow-up period was too short. Consequently, patients who survive longer are more likely to be defined as exposed, leading to an artificial protection of exposed patients.^13^ DDD indicates defined daily dose.

Despite extensive research on drug effectiveness and safety in schizophrenia, many observational studies have not accounted for immortal time bias (ITB), which can lead to overestimation of the protective association of treatment on mortality or underestimation of the harmful one due to study design.^11^ This bias may be particularly important when exposure is defined as having or not having received an intervention, such as surgery^12^ or drug treatment,^13,14^ at any time during follow-up. Immortal time bias refers to a period in the exposed group during which the outcome of interest cannot occur (Figure 1A).^11,14^ This immortal time is the lag between the start of follow-up and the beginning of exposure during which patients are necessarily alive and misclassified as exposed. This bias creates a scenario in which the exposed group appears to have a lower mortality rate than the unexposed group. Although widely recognized in the literature,^11,12,13,14^ this bias is not always addressed, potentially leading to inaccurate conclusions about the association between exposure and outcome.^14^

The present study had 2 aims. The first was to replicate previous study designs^7,8,9^ to assess whether cumulative doses of antipsychotics, antidepressants, and benzodiazepines are associated with mortality risk in patients with schizophrenia in Québec, Canada. The second was to assess the impact of not correcting for ITB and to suggest known solutions for dealing with this bias.

## Methods

This study was approved by the research ethics board committee of the Université de Sherbrooke and by the Commission d’Accès à l’Information du Québec. Informed consent is not required for registry-based studies using deidentified data. This study follows the Strengthening the Reporting of Observational Studies in Epidemiology (STROBE) reporting guideline for cohort studies.

### Design and Data Sources

This was a retrospective, population-based cohort study using administrative data from January 1, 2002, to December 31, 2017. In Québec, health care and social services are free and accessible to all residents. The hospital discharge register includes hospitalization dates, interventions, and principal and secondary diagnoses. The demographic database includes age, sex, date of death, and eligibility for the public drug insurance plan. The physician claims database provides the date, diagnosis, and service provided. The drug database contains information on the drugs claimed from community pharmacies by individuals covered by the public plan (outpatient care only), which includes about 90% of individuals aged 65 years or older, social assistance recipients, and those without private plans.

### Cohort Selection 

From these data, we extracted all patients diagnosed with schizophrenia (*International Classification of Diseases, Ninth Revision*: code 295; *International Statistical Classification of Diseases and Related Health Problems, Tenth Revision*: codes F20, F21, F23.2, and F25) between January 1, 2002, and December 31, 2012 (eFigure 1 in Supplement 1). Patients were considered to be diagnosed with schizophrenia if they had at least 1 hospitalization with a primary or secondary diagnosis of schizophrenia or at least 2 medical services within 2 years with 30 days or more of a gap between each claim with a diagnosis of schizophrenia. The follow-up period was January 1, 2013, until December 31, 2017, or death. Only patients aged 17 to 64 years at the beginning of follow-up were selected. All patients not continuously covered by public drug insurance 2 years before and during the follow-up period were excluded to ensure adequate measurement of drug exposure. Patients with fewer than 30 days of follow-up were also excluded.

### Outcome and Covariables

The outcome was all-cause death within 5 years from the beginning of follow-up. Covariables assessed at baseline (2011-2012) included age; sex; social welfare status; time since first diagnosis of schizophrenia; diagnosis of personality disorder; substance use disorders; previous use of antipsychotics, antidepressants, benzodiazepines and other anxiolytics, and mood stabilizers; 1 or more hospital admission for schizophrenia or psychosis; 1 or more hospital admission for another mental disorder; 1 or more hospital admission for a physical health reason; physical comorbidity index; and number of outpatient consultations.

### Drug Exposure

Exposure to antipsychotics, antidepressants, and benzodiazepines was defined separately as defined daily dose (DDD),^15^ with the mean determined over the outpatient follow-up days (excluding inpatient days), but not at baseline. Inpatient days were excluded due to the lack of data on drugs used during hospitalization. eTable 1 in Supplement 1 presents the list of DDDs used in the present study. For example, 1 unit of DDD corresponds to 10 mg of olanzapine, 100 mg of fluvoxamine, and 2.5 mg of lorazepam; hence, a 50% higher dose would correspond to 1.5 DDD. As with other studies,^7,8,16,17^ the 3 exposure variables were categorized according to no use (mean DDD = 0), small dosage or occasional use (0 < mean DDD < 0.5), moderate use (0.5 ≤ mean DDD ≤ 1.5), and high use (mean DDD >1.5). The period of drug exposure measure varies according to the statistical approach. A binary exposure variable was defined as no use or any use during follow-up.

### Statistical Analysis

Data analysis was performed from June 22, 2022, to September 30, 2024. Two approaches were used to treat exposure. The first approach used Cox proportional hazards regression models to examine the association between drugs and all-cause mortality. The treatment of exposure in Cox proportional hazards regression is central (eFigure 2 in Supplement 1). When drug exposure is static in a time-fixed model, the reference group is no exposure during follow-up (up to 5 years). With this approach, the whole period of follow-up is included in the mean DDD calculation. The suspected ITB arises because the time-fixed method assumes that a patient exposed to the drug (mean DDD > 0) remains exposed throughout follow-up, including the period between cohort entry and first prescription, during which death cannot occur by design (immortal time). This immortal time is misclassified as exposed (Figure 1).

The second approach was a Cox proportional hazards model with time-dependent drug exposure that controls for ITB and classifies the time before the first prescription as unexposed and the subsequent time as exposed. For the time-dependent method, the period included in the mean DDD calculation at time *t* is the period from time 0 to time *t* (Figure 1B).

All multivariable models were adjusted for covariates associated (*P* < .05) with all-cause death in a univariate analysis, after adjusting for age and sex. The 3 exposure variables were included. While the proportional hazards assumption is central to the Cox model, its importance is debated. Some authors interpret hazard ratios (HRs) as a mean association^18^ when this assumption is not met, while others stress its importance.^19,20^ For covariables not meeting the proportional hazards assumption, an interaction term with time was included in the model. However, for ease of interpretation, this interaction term was not included for drug exposure variables, considering the HR as a mean association. Adjusted HRs (AHRs) with 95% CIs and *P* values are reported, and we fixed the level of significance to *P* < .05 without correction for multiple comparisons. The characteristics of the study cohort were compared between groups using the Kruskal-Wallis test for continuous variables and the χ^2^ test for categorical variables. The results were generated using SAS, version 9.4 (SAS Institute Inc).

We performed 3 sensitivity analyses. First, as an alternative to the Cox proportional hazards model with time-dependent exposures, we performed a nested case-control design, where each death was associated with 5 matched controls randomly selected from the risk set. The case-control sample was then analyzed using a conditional logistic regression. Controls were matched to each case for age (±1 year), sex, comorbidity index (0, 1-2, and ≥3) and hospitalization for physical health reasons during the 2-year baseline period.

Second, to investigate whether results were similar in a more severe population, we repeated the analytic steps in a more severe subcohort, including only patients who had already been hospitalized for psychosis between 2002 and 2012. This subcohort may better reflect the cohort reported by Tiihonen et al.^7^

Third, since our definition of exposure does not take into account whether the patient was exposed at the time of death (for both the patient who died and the associated risk set, ie, all other cohort members who were still alive and at risk at that time), we also performed a current use analysis, with exposure to antipsychotics, antidepressants, and benzodiazepines defined as ongoing treatment (yes or no) in the week preceding death or in the week preceding the hospitalization leading to death.

## Results

### Characteristics of the Study Cohort

A total of 32 240 patients were included in the cohort (eFigure 1 in Supplement 1), with a mean (SD) age of 46.1 (11.6) years; 12 464 patients were women (38.7%) and 19 776 were men (61.3%) (Table). Characteristics based on time-fixed drug exposure levels (mean DDD) are reported in eTables 2-4 in Supplement 1. eFigure 3 in Supplement 1 shows the distribution of antipsychotic, antidepressant, or benzodiazepine use during follow-up: 31.8% of the cohort used all 3 types, and 5.4% used none. The mortality rate during follow-up was 6.0% (n = 1941) (Table).

**Table.** Characteristics of the Study Cohort by Antipsychotic, Antidepressant, and Benzodiazepine Binary Exposure During Follow-Up (N = 32 240)

Characteristic	Exposure, No. (%)									
	Total	No AP	At least 1 claim of AP	*P* value	No AD	At least 1 claim of AD	*P* value	No BZD	At least 1 claim of BZD	*P* value^a^
Total	32 240 (100)	2692 (8.3)	29 548 (91.7)	NA	16 841 (52.2)	15 399 (47.8)	NA	14 227 (44.1)	18 013 (55.9)	NA
Age, mean (SD), y	46.1 (11.6)	45.2 (11.9)	46.2 (11.6)	<.001	46.3 (11.5)	45.9 (11.8)	.04	44.6 (11.8)	47.3 (11.3)	<.001
Sex										
Female	12 464 (38.7)	988 (36.7)	11 476 (38.8)	.03	5622 (33.4)	6842 (44.4)	<.001	4781 (33.6)	7683 (42.6)	<.001
Male	19 776 (61.3)	1704 (63.3)	18 072 (61.2)		11 219 (66.6)	8557 (55.6)		9446 (66.4)	10 330 (57.4)	
Beneficiary status										
Social welfare with SRE	24 984 (77.5)	1277 (47.4)	23 707 (80.2)	<.001	12 988 (77.1)	11 996 (77.9)	.09	10 348 (72.7)	14 636 (81.2)	<.001
Other^b^	7256 (22.5)	1415 (52.6)	5841 (19.8)		3853 (22.9)	3403 (22.1)		3879 (27.3)	3377 (18.8)	
Time since first SCZ diagnosis										
In the last 2 y	1922 (6.0)	223 (8.3)	1699 (5.8)	<.001	856 (5.1)	1066 (6.9)	<.001	862 (6.1)	1060 (5.9)	<.001
Between 2 and 5 y	4347 (13.5)	588 (21.8)	3759 (12.7)		2034 (12.1)	2313 (15.0)		2052 (14.4)	2295 (12.7)	
Between 5 and 10 y	11 906 (36.9)	1215 (45.1)	10 691 (36.2)		6153 (36.5)	5753 (37.4)		5589 (39.3)	6317 (35.1)	
>10 y	14 065 (43.6)	666 (24.7)	13 399 (45.4)		7798 (46.3)	6267 (40.7)		5724 (40.2)	8341 (46.3)	
Characteristics measured during the baseline period (2011-2012)										
Personality disorder	4505 (14.0)	259 (9.6)	4246 (14.4)	<.001	1735 (10.3)	2770 (18.0)	<.001	1474 (10.4)	3031 (16.8)	<.001
Substance use disorder	5635 (17.5)	274 (10.2)	5361 (18.1)	<.001	2573 (15.3)	3062 (19.9)	<.001	2182 (15.3)	3453 (19.2)	<.001
Use of AP	28 862 (89.5)	514 (19.1)	28 461 (96.3)	<.001	14 676 (87.1)	14 299 (92.9)	<.001	12 039 (84.6)	16 936 (94.0)	<.001
Use of AD	13 088 (40.6)	700 (26.0)	12 388 (41.9)	<.001	1285 (7.6)	11 803 (76.6)	<.001	4156 (29.2)	8932 (49.6)	<.001
Use of BZD	16 023 (49.7)	626 (23.2)	15 397 (52.1)	<.001	6598 (39.2)	9425 (61.2)	<.001	1817 (12.8)	14 206 (78.9)	<.001
Use of mood stabilizers^c^	8692 (27.0)	258 (9.6)	8434 (28.5)	<.001	4290 (25.5)	4402 (28.6)	<.001	2980 (21.0)	5712 (31.7)	<.001
Hospitalization for psychosis	6367 (19.8)	152 (5.6)	6215 (21.0)	<.001	3358 (19.9)	3009 (19.5)	0.37	2533 (17.8)	3834 (21.3)	<.001
Hospitalization for other mental disorder	2976 (9.2)	132 (4.9)	2844 (9.6)	<.001	1102 (6.5)	1874 (12.2)	<.001	932 (6.6)	2044 (11.4)	<.001
Hospitalization for physical health	4731 (14.7)	375 (13.9)	4356 (14.7)	0.25	1990 (11.8)	2741 (17.8)	<.001	1620 (11.4)	3111 (17.3)	<.001
Comorbidity index										
0	23 765 (73.7)	2192 (81.4)	21 573 (73.0)	<.001	13 132 (78.0)	10 633 (69.0)	<.001	11 412 (80.2)	12 353 (68.6)	<.001
1-2	5690 (17.6)	328 (12.2)	5362 (18.2)		2564 (15.2)	3126 (20.3)		1988 (14.0)	3702 (20.6)	
≥3	2785 (8.6)	172 (6.4)	2613 (8.8)		1145 (6.8)	1640 (10.7)		827 (5.8)	1958 (10.9)	
No. of ambulatory visits, mean (SD)	18.7 (20.4)	11.2 (18.0)	19.4 (20.4)	<.001	15.9 (19.2)	21.8 (21.1)	<.001	15.4 (17.8)	21.4 (21.9)	<.001
Outcome										
All-cause death	1941 (6.0)	149 (5.5)	1792 (6.1)	.27	1068 (6.3)	873 (5.7)	.01	680 (4.8)	1261 (7.0)	<.001

Abbreviations: AD, antidepressant; AP, antipsychotic; BZD, benzodiazepine; NA, not applicable; SCZ, schizophrenia or schizoaffective disorder; SRE, severe restrictions on employment.

^a^ Kruskal-Wallis test (continuous variables); χ^2^ test (categorical variables).

^b^ Other includes beneficiaries with social welfare without SRE (n = 1567 [4.9%]) and regular beneficiaries (n = 5689 [17.6%]).

^c^ Including lithium, divalproex, lamotrigine, or carbamazepine.

### Antipsychotics and All-Cause Mortality

**Figure 2.**
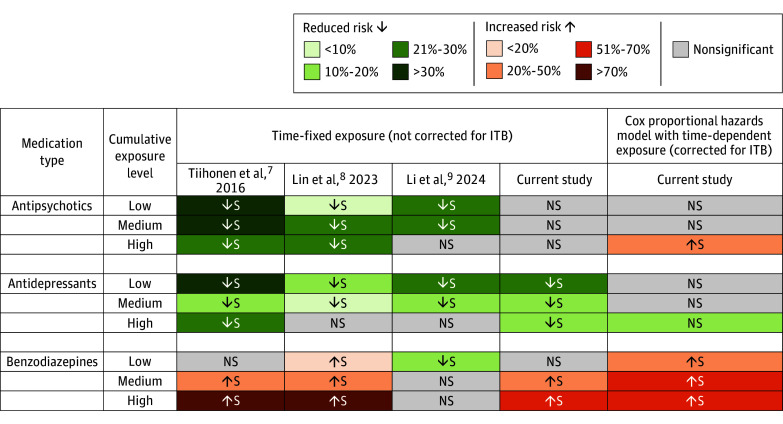
Comparative Results of the Risk Estimates for All-Cause Mortality Between Studies and Methods Cox proportional hazards regression model with time-fixed exposure not corrected for immortal time bias (ITB) and with time-dependent exposure corrected for ITB for antipsychotics, antidepressants, and benzodiazepines (N = 32 240). NS indicates nonsignificant; S, significant (*P* < .05).

**Figure 3.**
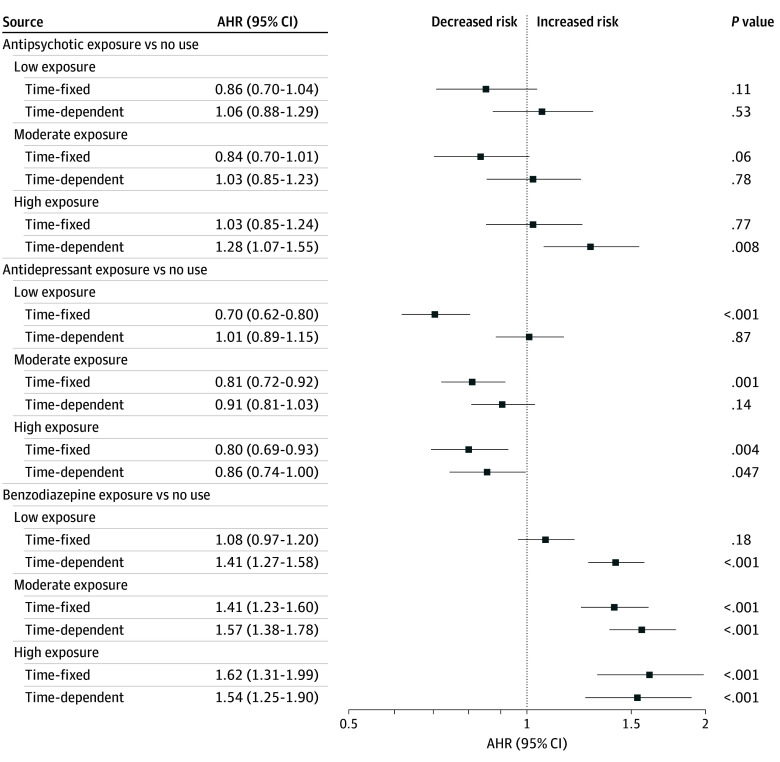
Adjusted Hazard Ratios (AHRs) for All-Cause Mortality for Cox Proportional Hazards Regression Models Time-fixed (not corrected for immortal time bias [ITB]) and time-dependent (corrected for ITB) exposure for antipsychotics, antidepressants, and benzodiazepines (N = 32 240). Error bars indicate 95% CIs.

The proportion of mortality was similar for antipsychotic users (6.1%) and nonusers (5.5%) (*P* = .27) (Table). Stratified by exposure levels, the mortality rate varied (nonuse: 5.5%, low: 5.8%, moderate: 5.4%, high: 7.0%; *P* = .001) (eTable 2 in Supplement 1). Compared with nonuse, any exposure to an antipsychotic during the follow-up was not associated with all-cause mortality in the adjusted analysis, correcting or not correcting for ITB (eTable 5A, eFigure 4 in Supplement 1). There was no dose-response association with mortality using the time-fixed method, but high-dose antipsychotic use was associated with increased mortality (AHR, 1.28; 95% CI, 1.07-1.55; *P* = .008) after correcting for ITB (Table, Figure 2, Figure 3; eTable 5B in Supplement 1).

### Antidepressants and All-Cause Mortality

Antidepressants users had a lower mortality rate (5.7%) compared with nonusers (6.3%) (*P* = .01) (Table), with rates increasing with dosage (low: 4.8%, moderate: 6.0%, high: 7.1%; *P* < .001) (eTable 3 in Supplement 1). Compared with nonuse, any exposure to antidepressants during the follow-up period was associated with better survival using the time-fixed method (AHR, 0.76; 95% CI, 0.70-0.84; *P* < .001) (eTable 5A, eFigure 4 in Supplement 1), but there was no association after controlling for ITB (AHR, 0.92; 95% CI, 0.84-1.01; *P* = .09). Regarding the dose-response association between antidepressants and mortality (Figures 2 and 3; eTable 5C in Supplement 1), not controlling for ITB resulted in a protective association for low (AHR, 0.70; 95% CI, 0.62-0.80; *P* < .001), moderate (AHR, 0.81; 95% CI, 0.72-0.92; *P* = .001), and high (AHR, 0.80; 95% CI, 0.69-0.93; *P* = .004) use, whereas only high use was associated with reduced mortality when we corrected for ITB (AHR, 0.86; 95% CI, 0.74-1.00; *P* = .047).

### Benzodiazepines and All-Cause Mortality

The mortality rate was higher among benzodiazepine users (7.0%) than nonusers (4.8%) (*P* < .001) (Table) and increased with dose (low: 6.0%, moderate: 9.6%, high: 12.5%; *P* < .001) (eTable 4 in Supplement 1). Benzodiazepine exposure was associated with a higher mortality risk (time-fixed: AHR, 1.22; 95% CI, 1.10-1.35; *P* < .001) (eTable 5A, eFigure 4 in Supplement 1), and this risk was greater with time-dependent exposure (AHR, 1.50; 95% CI, 1.36-1.65; *P* < .001). Mortality risk increased with doses, regardless of the method of analysis (Figure 2, Figure 3; eTable 5D in Supplement 1), and was higher when correcting for ITB.

### Sensitivity Analysis

The nested case-control design produced essentially the same results as the Cox proportional hazards model with time-dependent exposures (eTables 5-7, eFigures 4-7 in Supplement 1). In the more severe subcohort, any antipsychotic use during the follow-up, as well as in the low- and moderate-exposure groups, was found to be protective with the time-fixed approach, but was not associated with all-cause mortality when we corrected for ITB (eTables 6 and 7, eFigures 5 and 6 in Supplement 1). Results for antidepressants and benzodiazepines in the more-severe subcohort were similar to those reported in the whole cohort. In the current use analysis, the results revealed a reduced risk of mortality for antipsychotics (AHR, 0.68; 95% CI, 0.61-0.77; *P* < .001) and antidepressants (AHR, 0.90; 95% CI, 0.81-0.99; *P* = .03), and an increased risk of mortality for benzodiazepines (AHR, 1.30; 95% CI, 1.19-1.44; *P* < .001).

### Immortal Time Bias

Adjustment for ITB typically increased AHRs (ie, a decrease of the protective association for AHR <1 and an increase of the protective association for AHR ≥1) compared to time-fixed exposure methods. For antidepressants, the apparent protective association at lower doses was reduced after ITB adjustment (Figures 2 and 3; eTable 5C in Supplement 1). Benzodiazepine risk estimates remained consistent across exposure levels, with a notable increase in the low-dose group after ITB correction. No reduced mortality risk was found in the cumulative dose levels of antipsychotics with any method of analysis, but high-dose exposure was associated with increased mortality after ITB adjustment (Figures 2 and 3; eTable 5B in Supplement 1), which was not found when restricted to a more-severe cohort (eTables 6 and 7, eFigure 6 in Supplement 1).

## Discussion

**Figure 4.**
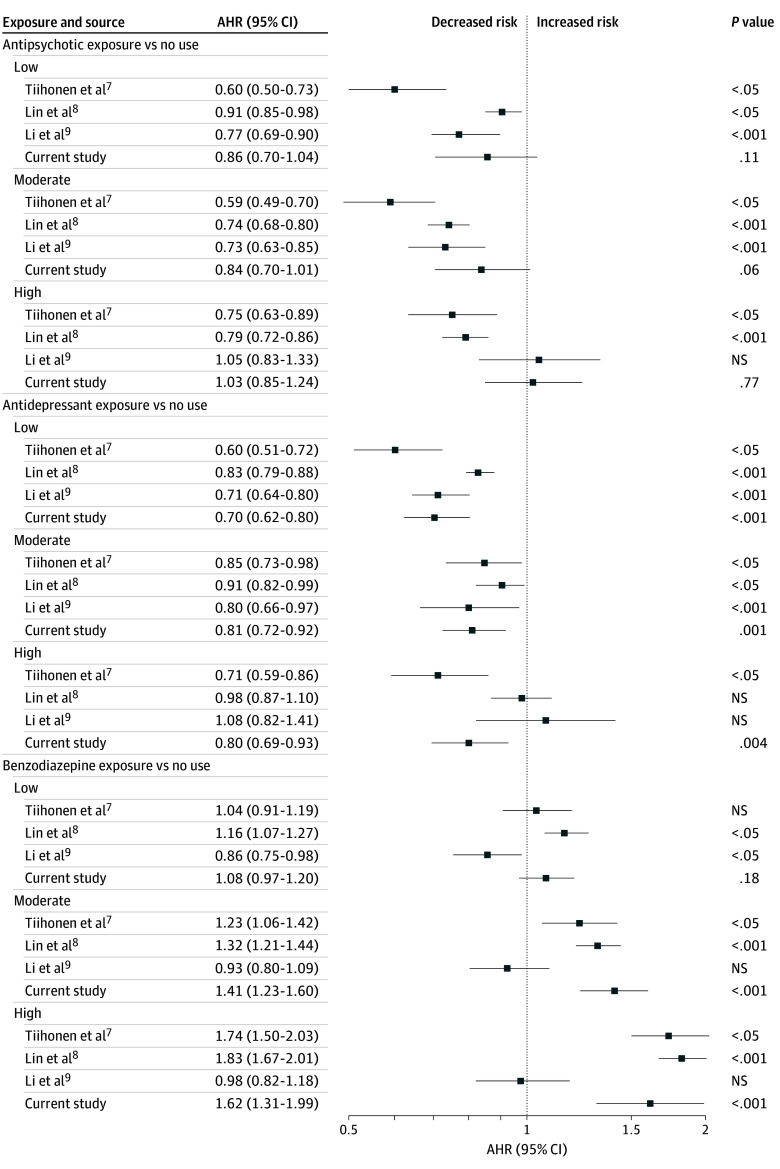
Comparison of Adjusted Hazard Ratios (AHRs) for All-Cause Mortality Findings shown for studies of Tiihonen et al^7^ (N = 21 492, not corrected for immortal time bias [ITB]), Lin et al^8^ (N = 102 964, not corrected for ITB), Li et al^9^ (N = 6433, not corrected for ITB), and the current study (N = 32 240, time-fixed method, not corrected for ITB). To compare similar methods, time-fixed exposure to antidepressants and benzodiazepines was used to report the results. NS indicates nonsignificant (*P* ≥ .05). Error bars indicate 95% CI.

In the present study, we first aimed to replicate the design, without controlling for ITB, of 3 large observational studies^7,8,9^ assessing whether the cumulative dose of antipsychotics, antidepressants, and benzodiazepines in the treatment of schizophrenia was associated with all-cause mortality in patients with schizophrenia. While these studies typically found a protective association of antipsychotics, we found no such association with mortality, whether correcting or not correcting for ITB (Figures 2 and 4). These studies also found a reduced mortality risk for antidepressants, as we did when not controlling for ITB, but the advantage was not found after controlling for it. Benzodiazepines were generally associated with an increased risk of mortality, whether controlling or not controlling for ITB, and this was consistent with other studies,^6,7,8,21,22^ but not in an older population.^9^

The impact of not controlling for ITB, a well-documented issue in medical research,^11,12,13,14,23,24,25^ confirmed that it tends to overestimate the protective association of drugs or underestimate the harmful ones and reinforces the need for robust analytical techniques that account for ITB. Our findings regarding antipsychotic use and all-cause mortality (no association) contrast with those reported in 2 meta-analyses conducted in 2017,^26^ and 2022.^2^ In meta-analyzing a subset of 4 large, retrospective cohort studies,^16,27,28,29^ Vermeulen et al^26^ found a reduced risk of death in patients who had received some antipsychotic vs no antipsychotic treatment (risk ratios ranging from 0.48 to 0.63; global risk ratio, 0.59). However, 3^16,27,28^ of the 4 studies did not control for ITB, and thus may have overestimated the protective association of antipsychotics. The ITB control status in the fourth study was unclear.^29^ Hence, the difference between our results and those reported by Vermeulen et al^26^ may be at least partly explained by the correction for ITB. The 2022 meta-analysis^2^ also found a protective association of antipsychotics with mortality (global relative risk, 0.71) based on 11 observational studies. A closer look at these studies reveals that some of them^27,30^ did not correct for ITB. For example, Oh et al,^30^ who included 77 139 patients with schizophrenia, classified patients as antipsychotic-treated if they had at least 4 weeks of exposure during a 15-year follow-up, ignoring the immortal time period, introducing bias. However, some of studies reported by Correll et al,^2^ in particular a large cohort study of 62 250 patients with schizophrenia,^5^ that controlled for ITB, found a reduced risk of mortality associated with antipsychotics.

Other factors may explain discrepancies between our results and those reported in the literature.^2,26^ First, the severity of schizophrenia in the cohorts studied varies; in our more-severe subcohort, the results were consistent with the findings of Tiihonen et al^7^ without ITB control, indicating lower mortality with any antipsychotic use (eFigure 5 in Supplement 1). After correction for ITB, no association was found. The crude mortality rates between the unexposed and exposed antipsychotic groups in our study (5.5% vs 6.1%) (Table) differ from those in Tiihonen et al,^7^ suggesting that the unexposed group in the Tiihonen et al^7^ study was more severely ill or at higher risk of death.

Second, the design and definition of exposure differ between studies. We defined exposure as a mean daily dose exposure over the whole outpatient follow-up, while other studies^5,31^ defined exposure as ongoing exposure at the time of death. However, when we performed such a current use analysis, our results were more in line with the literature. An explanation of the discrepancy between our cumulative exposure and current use analyses may be the difference in the reference group under study, which has implications for the HR reported. In the first case, the nonexposure group refers to patients without treatment for the whole follow-up period, while the nonexposure group in the current use analyses refers to those not receiving treatment around the time of death, either because they discontinued their treatment or they had not used the treatment.

Despite the mixed results on mortality, it is important to recognize the established benefits of antipsychotics in the treatment of schizophrenia. Our findings do not undermine the potential benefit of antipsychotic treatment; rather, they call for a reevaluation of the methods used to assess these effects. The current study, together with evidence from previous research supporting the usefulness of antipsychotics in the treatment of schizophrenia,^31^ suggests that although antipsychotics have well-documented benefits, the assessment of their association with mortality requires careful consideration of dosage, patient characteristics, and methods of analysis. By incorporating appropriate methods that account for biases such as ITB, future research can provide clearer insights into the true effects of psychiatric medications in different types of populations.

### Limitations

This study has limitations. Only patients with schizophrenia whose drug treatment is covered by the public drug plan were included. However, the risk of selection bias is minimized by including all patients diagnosed with schizophrenia, almost 80% of whom receive services covered by public drug insurance.^31^ Residual information bias cannot be excluded because the data used are initially collected for administrative purposes and not for research (ie, some useful information may be missing; eg, severity of illness and inpatient drug use).

## Conclusions

In this cohort study of 32 240 patients with schizophrenia, we examined the use of cumulative doses of antipsychotics, antidepressants, and benzodiazepines and all-cause mortality in patients with schizophrenia using 2 approaches: with and without ITB control. Contrary to previous research suggesting a protective association of antipsychotics and antidepressants, our results showed no such association when controlling for ITB. However, benzodiazepines were consistently associated with increased mortality regardless of ITB control. These findings suggest that not adjusting for ITB may overestimate the protective association of these drugs with mortality and highlight the need for appropriate analytical techniques.

## Notes

Supplement 1. **eTable 1.** Defined Daily Doses From the World Health Organization Collaborating Center for Drug Statistics Methodology **eTable 2.** Characteristics of the Study Cohort by Antipsychotics (AP) Time-Fixed Exposure Category (n = 32,240) **eTable 3.** Characteristics of the Study Cohort by Antidepressants (AD) Time-Fixed Exposure Category (n = 32,240) **eTable 4.** Characteristics of the Study Cohort by Benzodiazepines (BZD) Time-Fixed Exposure Category (n = 32,240) **eTable 5.** Crude and Adjusted Hazard Ratios (HR) for Death According to Time-Fixed vs Time- Dependent Exposure to Levels of Antipsychotics, Antidepressants, and Benzodiazepines (n = 32,240) **eTable 6.** Adjusted Hazard Ratios for All-Cause Mortality According to Time-Fixed vs Time- Dependent Exposure to Levels of Antipsychotics, Antidepressants, and Benzodiazepines in the More Severe Sub-Cohort (n = 18,584) **eTable 7.** Comparative Results of the Risk Estimates for All-Cause Mortality Between Studies and Methods: Time-Fixed Exposure (Not Corrected for ITB) and Time-Dependent Exposure (Corrected for ITB, Cox and NCC) for Antipsychotics, Antidepressants, and Benzodiazepines (More Severe Sub-Cohort, n = 18,584) **eFigure 1.** Selection of the Study Cohort (n = 32,240) **eFigure 2.** Design of the Study Cohort (n = 32,240) **eFigure 3.** Distribution of the Study Cohort According to Any Use of Antipsychotics, Antidepressants, or Benzodiazepines During Follow-Up **eFigure 4.** Adjusted Hazard Ratios (HR) for All-Cause Mortality for Time-Fixed (Not Corrected for ITB) and Time-Dependent Exposure (Corrected for ITB, Cox and NCC) for Binary Exposure to Antipsychotics, Antidepressants, and Benzodiazepines (n = 32,240) **eFigure 5.** Adjusted Hazard Ratios (HR) for All-Cause Mortality for Time-Fixed (Not Corrected for ITB) and Time-Dependent Exposure (Corrected for ITB, Cox and NCC) for Binary Exposure to Antipsychotics, Antidepressants, and Benzodiazepines (More Severe Sub-Cohort, n = 18,584) **eFigure 6.** Adjusted Hazard Ratios (HR) for All-Cause Mortality for Time-Fixed (Not Corrected for ITB) and Time-Dependent Exposure (Corrected for ITB, Cox and NCC) for Antipsychotics, Antidepressants, and Benzodiazepines (More Severe Sub-Cohort, n = 18,584) **eFigure 7.** Comparison of Adjusted Hazard Ratios (HR) for All-Cause Mortality of Tiihonen et al. (2016, n = 21,492, Not Corrected for ITB)^7^, Lin et al. (2023, n = 102,964, Not Corrected for ITB)^8^, Li et al. (2024, n = 6433, Not Corrected for ITB)^9^, and the Current Study (More Severe Sub-Cohort, n = 18,584, Time-Fixed Method, Not Corrected for ITB)

Supplement 2.
Data Sharing Statement
