# Supplementary material for: Medication Exposure and Mortality in Patients With Schizophrenia
Source: JAMA Netw Open. 2024 Nov 22;7(11):e2447137. doi: 10.1001/jamanetworkopen.2024.47137 (PMC11584925; doi:10.1001/jamanetworkopen.2024.47137)
Supplement: Supplement 1. — eTable 1. Defined Daily Doses From the World Health Organization Collaborating Center for Drug Statistics Methodology eTable 2. Characteristics of the Study Cohort by Antipsychotics (AP) Time-Fixed Exposure Category (n = 32,240) eTable 3. Characteristics of the Study Cohort by Antidepressants (AD) Time-Fixed Exposure Category (n = 32,240) eTable 4. Characteristics of the Study Cohort by Benzodiazepines (BZD) Time-Fixed Exposure Category (n = 32,240) eTable 5. Crude and Adjusted Hazard Ratios (HR) for Death According to Time-Fixed vs Time- Dependent Exposure to Levels of Antipsychotics, Antidepressants, and Benzodiazepines (n = 32,240) eTable 6. Adjusted Hazard Ratios for All-Cause Mortality According to Time-Fixed vs Time- Dependent Exposure to Levels of Antipsychotics, Antidepressants, and Benzodiazepines in the More Severe Sub-Cohort (n = 18,584) eTable 7. Comparative Results of the Risk Estimates for All-Cause Mortality Between Studies and Methods: Time-Fixed Exposure (Not Corrected for ITB) and Time-Dependent Exposure (Corrected for ITB, Cox and NCC) for Antipsychotics, Antidepressants, and Benzodiazepines (More Severe Sub-Cohort, n = 18,584) eFigure 1. Selection of the Study Cohort (n = 32,240) eFigure 2. Design of the Study Cohort (n = 32,240) eFigure 3. Distribution of the Study Cohort According to Any Use of Antipsychotics, Antidepressants, or Benzodiazepines During Follow-Up eFigure 4. Adjusted Hazard Ratios (HR) for All-Cause Mortality for Time-Fixed (Not Corrected for ITB) and Time-Dependent Exposure (Corrected for ITB, Cox and NCC) for Binary Exposure to Antipsychotics, Antidepressants, and Benzodiazepines (n = 32,240) eFigure 5. Adjusted Hazard Ratios (HR) for All-Cause Mortality for Time-Fixed (Not Corrected for ITB) and Time-Dependent Exposure (Corrected for ITB, Cox and NCC) for Binary Exposure to Antipsychotics, Antidepressants, and Benzodiazepines (More Severe Sub-Cohort, n = 18,584) eFigure 6. Adjusted Hazard Ratios (HR) for All-Cause Mortality fo [file jamanetwopen-e2447137-s001.pdf]

Supplementary Online Content

Brodeur S, Chiu YM, Courteau J, et al. Medication exposure and mortality in patients with schizophrenia. *JAMA Netw Open*. 2024;7(11):e2447137.
doi:10.1001/jamanetworkopen.2024.47137

eTable 1. Defined Daily Doses From the World Health Organization Collaborating Center for Drug Statistics Methodology

eTable 2. Characteristics of the Study Cohort by Antipsychotics (AP) Time-Fixed Exposure Category (n = 32,240)

eTable 3. Characteristics of the Study Cohort by Antidepressants (AD) Time-Fixed Exposure Category (n = 32,240)

eTable 4. Characteristics of the Study Cohort by Benzodiazepines (BZD) Time-Fixed Exposure Category (n = 32,240)

eTable 5. Crude and Adjusted Hazard Ratios (HR) for Death According to Time-Fixed vs. Time-Dependent Exposure to Levels of Antipsychotics, Antidepressants, and Benzodiazepines (n = 32,240)

eTable 6. Adjusted Hazard Ratios for All-Cause Mortality According to Time-Fixed vs. Time-Dependent Exposure to Levels of Antipsychotics, Antidepressants, and Benzodiazepines in the More Severe Sub-Cohort (n = 18,584)

eTable 7. Comparative Results of the Risk Estimates for All-Cause Mortality Between Studies and Methods: Time-Fixed Exposure (Not Corrected for ITB) and Time-Dependent Exposure (Corrected for ITB, Cox and NCC) for Antipsychotics, Antidepressants, and Benzodiazepines (More Severe Sub-Cohort, n=18,584)

eFigure 1. Selection of the Study Cohort (n=32,240) **eFigure 2.** Design of the Study Cohort (n=32,240)

eFigure 3. Distribution of the Study Cohort According to Any Use of Antipsychotics, Antidepressants, or Benzodiazepines During Follow-Up

eFigure 4. Adjusted Hazard Ratios (HR) for All-Cause Mortality for Time-Fixed (Not Corrected for ITB) and Time-Dependent Exposure (Corrected for ITB, Cox and NCC) for Binary Exposure to Antipsychotics, Antidepressants, and Benzodiazepines (n=32,240) **eFigure 5.** Adjusted Hazard Ratios (HR) for All-Cause Mortality for Time-Fixed (Not Corrected for ITB) and Time-Dependent Exposure (Corrected for ITB, Cox and NCC) for Binary Exposure to Antipsychotics, Antidepressants, and Benzodiazepines (More Severe Sub-Cohort, n=18,584)

eFigure 6. Adjusted Hazard Ratios (HR) for All-Cause Mortality for Time-Fixed (Not Corrected for ITB) and Time-Dependent Exposure (Corrected for ITB, Cox and NCC) for Antipsychotics, Antidepressants, and Benzodiazepines (More Severe Sub-Cohort, n=18,584)

eFigure 7. Comparison of Adjusted Hazard Ratios (HR) for All-Cause Mortality of Tiihonen et al. (2016, n=21,492, Not Corrected for ITB)[7], Lin et al. (2023, n=102,964, Not Corrected for ITB)[8], Li et al. (2024, n=6433, Not Corrected for ITB)[9], and the Current Study (More Severe Sub-Cohort, n=18,584, Time-Fixed Method, Not Corrected for ITB)

This supplementary material has been provided by the authors to give readers additional information about their work.

© 2024 Brodeur S et al. *JAMA Network Open.*

eTable 1. Defined daily doses from the World Health Organization collaborating center for drug statistics methodology

Antipsychotic	Anatomical therapeutic class (ATC)	Route of Administration	DDD (mg)
CHLORPROMAZINE	N05AA01	O,R (P)	300 (100)
DROPERIDOL	N05AD08	P	2.5
FLUPENTHIXOL	N05AF01	O (P)	6 (4)
FLUPHENAZINE	N05AB02	O (P)	10 (1)
FLUSPIRILENE	N05AG01	P	0.7
HALOPERIDOL	N05AD01	O (P)	8 (3.3)
LOXAPINE	N05AH01	O	100
MESORIDAZINE	N05AC03	O,P	200
METHOTRIMEPRAZINE	N05AA02	O (P)	300 (100)
PERICYAZINE	N05AC01	O (P)	50 (20)
PERPHENAZINE	N05AB03	O (P)	30 (7)
PIMOZIDE	N05AG02	O	4
PIPOTIAZINE	N05AC04	O (P)	10 (5)
PROCHLORPERAZINE	N05AB04	O,R (P)	100 (50)
PROMAZINE	N05AA03	O (P)	300 (100)
THIOPROPAZATE	N05AB05	O	60
THIOPROPERAZINE	N05AB08	O (P)	75 (20)
THIORIDAZINE	N05AC02	O	300
THIOTHIXENE	N05AF04	O	30
TRIFLUOPERAZINE	N05AB06	O,R (P)	20 (8)
TRIFLUOPROMAZINE	N05AA05	O,P	100
ZUCLOPENTHIXOL	N05AF05	O (P)	30 (15)
ARIPIPRAZOLE	N05AX12	O (P)	15 (13.3)
ASENAPINE	N05AH05	O	400
BREXIPIPRAZOLE	N05AX16	O	3
CLOZAPINE	N05AH02	O,P	300
LURASIDONE	N05AE05	O	60
OLANZAPINE	N05AH03	O,P	10
PALIPERIDONE	N05AX13	O (P)	6 (2.5)
QUETIAPINE	N05AH04	O	400
RISPERIDONE	N05AX08	O (P)	5 (2.7)
ZIPRASIDONE	N05AE04	O (P)	80 (40)
**Antidepressant**	**Anatomical therapeutic class (ATC)**	**Route of Administration**	**DDD (mg)**
AMITRIPTYLINE	N06AA09	O, P	75
BUPROPION	N06AX12	O	300
CITALOPRAM	N06AB04	O, P	20

© 2024 Brodeur S et al. *JAMA Network Open.*

CLOMIPRAMINE	N06AA04	O, P	100
DESIPRAMINE	N06AA01	O	100
DESVENLAFAXINE	N06AX23	O	50
DOXEPIN	N06AA12	O, P	100
DULOXETINE	N06AX21	O	60
ESCITALOPRAM	N06AB10	O	10
FLUVOXAMINE	N06AB08	O	100
FLUOXETINE	N06AB03	O	20
IMIPRAMINE	N06AA02	O, P	100
LEVOMILNACIPRAN	N06AX28	O	40
NORTRIPTYLINE	N06AA10	O	75
MIRTAZAPINE	N06AX11	O	30
MOCLOBEMIDE	N06AC02	O	300
PAROXETINE	N06AB05	O	20
PHENELZINE	N06AF04	O	60
SERTRALINE	N06AB06	O	50
TRANYLCYPROMINE	N06AF04	O	10
TRAZODONE	N06AX05	O	300
TRIMIPRAMINE	N06AA06	O, P	150
VENLAFAXINE	N06AX16	O	100
VORTIOXETINE	N06AX26	O	10
**Benzodiazepine and other anxiolytics**	**ATC**	**Route of Administration**	**DDD (mg)**
ALPRAZOLAM	N05BA12	O	1
BROMAZEPAM	N05BA08	O	10
CLOBAZAM	N05BA09	O	20
CLONAZEPAM	N03AE01	O, P	8
CHLORDIAZEPOXIDE	N05BA02	O	30
DIAZEPAM	N05BA01	O, P, R	10
FLURAZEPAM	N05CD01	O	30
LORAZEPAM	N05BA06	O, P	2.5
MIDAZOLAM	N05CD08	O, P	15
NITRAZEPAM	N05CD02	O	5
OXAZEPAM	N05BA04	O	50
TEMAZEPAM	N05CD07	O	20
ZOLPIDEM	N05CF02	O	10
ZOPICLONE	N05CF01	O	7.5

Route of Administration: O – Oral, P – Percutaneous, R – Rectal.

© 2024 Brodeur S et al. *JAMA Network Open.*

eTable 2. Characteristics of the study cohort by antipsychotics (AP) time-fixed exposure category (n = 32,240)

	No AP	Low dose or occasional AP users	Moderate AP doses	High AP doses	p-value*
Total, n (%)	2692 (8.3)	6262 (19.4)	12,368 (38.4)	10,918 (33.9)	-
Age, mean (SD)	45.2 (11.9)	47.1 (11.8)	46.0 (11.6)	45.9 (11.4)	<.001
Sex, n (%)					<.001
Female	988 (36.7)	3065 (48.9)	4982 (40.3)	3429 (31.4)	
Male	1704 (63.3)	3197 (51.1)	7386 (59.7)	7489 (68.6)	
Beneficiary status, n (%)					<.001
Social welfare with SRE	1277 (47.4)	3998 (63.8)	9909 (80.1)	9800 (89.8)	
Other	1415 (52.6)	2264 (36.2)	2459 (19.9)	1118 (10.2)	
Time since first SCZ diagnosis, n (%)					<.001
In the last 2 years	223 (8.3)	578 (9.2)	749 (6.1)	372 (3.4)	
Between 2 and 5 years	588 (21.8)	1122 (17.9)	1663 (13.4)	974 (8.9)	
Between 5 and 10 years	1215 (45.1)	2574 (41.1)	4558 (36.9)	3559 (32.6)	
More than 10 years	666 (24.7)	1988 (31.8)	5398 (43.6)	6013 (55.1)	
**Characteristics measured during the baseline period (2011-2012)**					
Personality disorder, n (%)	259 (9.6)	1034 (16.5)	1725 (13.9)	1487 (13.6)	<.001
Substance use disorder, n (%)	274 (10.2)	1058 (16.9)	2153 (17.4)	2150 (19.7)	<.001
Use of AP, n (%)	514 (19.1)	5503 (87.9)	12,141 (98.2)	10,817 (99.1)	<.001
Use of AD, n (%)	700 (26.0)	2939 (46.9)	5129 (41.5)	4320 (39.6)	<.001
Use of BZD, n (%)	626 (23.3)	2878 (46.0)	6170 (49.9)	6349 (58.2)	<.001
Use of Mood Stabilisers†, n (%)	258 (9.6)	1479 (23.6)	3439 (27.8)	3516 (32.2)	<.001
Hosp. psychosis, n (%)	152 (5.6)	684 (10.9)	2461 (19.9)	3070 (28.1)	<.001
Hosp. other mental dis., n (%)	132 (4.9)	706 (11.3)	1229 (9.9)	909 (8.3)	<.001
Hosp. physical health, n (%)	375 (13.9)	1049 (16.8)	1742 (14.1)	1565 (14.3)	<.001
Comorbidity index, n (%)					<.001
0	2192 (81.4)	4524 (72.2)	9138 (73.9)	7911 (72.5)	
1-2	328 (12.2)	1156 (18.5)	2190 (17.7)	2016 (18.5)	
≥ 3	172 (6.4)	582 (9.3)	1040 (8.4)	991 (9.1)	
Number of ambulatory visits, mean (SD)	11.2 (18.0)	18.4 (21.7)	19.1 (19.8)	20.4 (20.3)	<.001
**Outcome**					
All-cause death, n (%)	149 (5.5)	363 (5.8)	670 (5.4)	759 (7.0)	<.001

Abbreviations: AP: antipsychotics; SCZ: schizophrenia or schizoaffective disorder; SD: standard deviation; SRE: severe restrictions on employment disorder; AD: antidepressants; BZD: benzodiazepines. Kruskal-Wallis test (continuous variables); Khi-2 test (categorical variables).

* Kruskal-Wallis test (continuous variables); Khi-2 test (categorical variables)

† Including lithium, divalproex, lamotrigine or carbamazepine

© 2024 Brodeur S et al. *JAMA Network Open.*

eTable 3. Characteristics of the study cohort by antidepressants (AD) time-fixed exposure category (n = 32,240)

	No AD	Low dose or occasional AD users	Moderate AD doses	High AD doses	p-value*
Total, n (%)	16,841 (52.2)	6298 (19.5)	5805 (18.0)	3296 (10.2)	-
Age, mean (SD)	46.3 (11.5)	44.2 (12.2)	46.8 (11.4)	47.7 (11.0)	<.001
Sex, n (%)					<.001
Female	5622 (33.4)	2540 (40.3)	2715 (46.8)	1587 (48.2)	
Male	11,219 (66.6)	3758 (59.7)	3090 (53.2)	1709 (51.8)	
Beneficiary status, n (%)					<.001
Social welfare with SRE	12,988 (77.1)	4756 (75.5)	4577 (78.8)	2663 (80.8)	
Other	3853 (22.9)	1542 (24.5)	1228 (21.2)	633 (19.2)	
Duration of SCZ, n (%)					<.001
In the last 2 years	856 (5.1)	478 (7.6)	375 (6.5)	213 (6.5)	
Between 2 and 5 years	2034 (12.1)	992 (15.8)	834 (14.4)	487 (14.8)	
Between 5 and 10 years	6153 (36.5)	2339 (37.1)	2139 (36.8)	1275 (38.7)	
More than 10 years	7798 (46.3)	2489 (39.5)	2457 (42.3)	1321 (40.1)	
**Characteristics measured during the baseline period (2011-2012)**					
Personality disorder, n (%)	1735 (10.3)	1153 (18.3)	969 (16.7)	648 (19.7)	<.001
Substance use disorder, n (%)	2573 (15.3)	1526 (24.2)	991 (17.1)	545 (16.5)	<.001
Use of AP, n (%)	14,609 (86.8)	5737 (91.1)	5455 (94.0)	3107 (94.3)	<.001
Use of AD, n (%)	1285 (7.6)	3405 (54.1)	5196 (89.5)	3202 (97.2)	<.001
Use of BZD, n (%)	6598 (39.2)	3530 (56.0)	3585 (61.8)	2310 (70.1)	<.001
Use of Mood Stabilisers[b], n (%)	4290 (25.5)	1843 (29.3)	1651 (28.4)	908 (27.6)	<.001
Hosp. psychosis, n (%)	3358 (19.9)	1542 (24.5)	1011 (17.4)	456 (13.8)	<.001
Hosp. other mental dis., n (%)	1102 (6.5)	821 (13.0)	639 (11.0)	414 (12.6)	<.001
Hosp. physical health, n (%)	1990 (11.8)	966 (15.3)	1079 (18.6)	696 (21.1)	<.001
Comorbidity index, n (%)					<.001
0	13,132 (78.0)	4492 (71.3)	3989 (68.7)	2152 (65.3)	
1-2	2564 (15.2)	1239 (19.7)	1176 (20.3)	711 (21.6)	
≥ 3	1145 (6.8)	567 (9.0)	640 (11.0)	433 (13.1)	
Number of ambulatory visits, mean (SD)	15.9 (19.2)	21.7 (22.2)	21.3 (20.4)	22.8 (20.1)	<.001
**Outcome**					
All-cause death, n (%)	1068 (6.3)	293 (4.6)	358 (6.2)	222 (6.7)	<.001

Abbreviations: AP: antipsychotics; SCZ: schizophrenia or schizoaffective disorder; SD: standard deviation; SRE: severe restrictions on employment disorder; AD: antidepressants; BZD: benzodiazepines. Kruskal-Wallis test (continuous variables); Khi-2 test (categorical variables).

[a] Deprived area means an area being in the most deprived quintile; 3351 missing values for variables materially and socially deprived area;

[b] Including lithium, divalproex, lamotrigine or carbamazepine

* Kruskal-Wallis test (continuous variables); Khi-2 test (categorical variables)

© 2024 Brodeur S et al. *JAMA Network Open.*

eTable 4. Characteristics of the study cohort by benzodiazepines (BZD) time-fixed exposure category (n = 32,240)

	No BZD	Low dose or occasional BZD users	Moderate BZD doses	High BZD doses	p-value*
Total, n (%)	14,227 (44.1)	12,870 (39.9)	4215 (13.1)	928 (2.9)	-
Age, mean (SD)	44.6 (11.8)	46.1 (11.6)	50.2 (9.9)	50.9 (9.1)	<.001
Sex, n (%)					<.001
Female	4781 (33.6)	5497 (42.7)	1801 (42.7)	385 (41.5)	
Male	9446 (66.4)	7373 (57.3)	2414 (57.3)	543 (58.5)	
Beneficiary status, n (%)					<.001
Social welfare with SRE	10,348 (72.7)	10,184 (79.1)	3634 (86.2)	818 (88.2)	
Other	3879 (27.3)	2686 (20.9)	581 (13.8)	110 (11.8)	
Duration of SCZ, n (%)					<.001
In the last 2 years	862 (6.1)	845 (6.6)	177 (4.2)	38 (4.1)	
Between 2 and 5 years	2052 (14.4)	1732 (13.5)	445 (10.6)	118 (12.7)	
Between 5 and 10 years	5589 (39.3)	4604 (35.8)	1382 (32.8)	331 (35.7)	
More than 10 years	5724 (40.2)	5689 (44.2)	2211 (52.5)	441 (47.5)	
**Characteristics measured during the baseline period (2011-2012)**					
Personality disorder, n (%)	1474 (10.4)	2205 (17.1)	662 (15.7)	164 (17.7)	<.001
Substance use disorder, n (%)	2182 (15.3)	2587 (20.1)	700 (16.6)	166 (17.9)	<.001
Use of AP, n (%)	12,039 (84.6)	11,982 (93.1)	4069 (96.5)	885 (95.4)	<.001
Use of AD, n (%)	4156 (29.2)	6206 (48.2)	2192 (52.0)	534 (57.5)	<.001
Use of BZD, n (%)	1817 (12.8)	9253 (71.9)	4031 (95.6)	922 (99.4)	<.001
Use of Mood Stabilisers[b], n (%)	2980 (21.0)	4135 (32.1)	1309 (31.6)	268 (28.9)	<.001
Hosp. psychosis, n (%)	2533 (17.8)	2957 (23.0)	755 (17.9)	122 (13.2)	<.001
Hosp. other mental dis., n (%)	932 (6.6)	1544 (12.0)	400 (9.5)	100 (10.8)	<.001
Hosp. physical health, n (%)	1620 (11.4)	2093 (16.3)	808 (19.2)	210 (22.6)	<.001
Comorbidity index, n (%)					<.001
0	11,412 (80.2)	9039 (70.2)	2733 (64.8)	581 (62.6)	
1-2	1988 (14.0)	2564 (19.9)	940 (22.3)	198 (21.3)	
≥ 3	827 (5.8)	1267 (9.8)	542 (12.9)	149 (16.1)	
Number of ambulatory visits, mean (SD)	15.4 (17.8)	21.5 (21.3)	20.9 (23.4)	21.8 (22.6)	<.001
**Outcome**					
All-cause death, n (%)	680 (4.8)	751 (5.8)	400 (9.5)	110 (11.9)	<.001

Abbreviations: AP: antipsychotics; SCZ: schizophrenia or schizoaffective disorder; SD: standard deviation; SRE: severe restrictions on employment disorder; SD: standard deviation; AD: antidepressants; BZD: benzodiazepines
[a] Deprived area means an area being in the most deprived quintile; 3351 missing values for variables materially and socially deprived area;
[b] Including lithium, divalproex, lamotrigine or carbamazepine

* Kruskal-Wallis test (continuous variables); Khi-2 test (categorical variables)

© 2024 Brodeur S et al. *JAMA Network Open.*

eTable 5. Crude and adjusted[*] hazard ratios (HR) for death according to time-fixed vs. time-dependent exposure to levels of antipsychotics, antidepressants, and benzodiazepines (n = 32,240)

A

	**Any exposure during follow-up (5 years for survivors; until death if deceased)**					
	**Time-fixed exposure (Cox)**		**Time-dependent exposure (Cox)**		**Time-dependent exposure (NCC)**	
	Crude HR (95% CI)	Adjusted HR (95% CI)	Crude HR (95% CI)	Adjusted HR (95% CI)	Crude HR (95% CI)	Adjusted HR (95% CI)
Antipsychotics	1.09 (0.92 - 1.29) p=0.30	0.91 (0.76 - 1.08)[†] p=0.27	**1.43 (1.21 - 1.69) p<.001**	0.92 (0.77 - 1.10) p=0.36	**1.32 (1.10 - 1.58) p=0.003**	1.18 (0.98 - 1.42) p=0.09
Antidepressants	**0.89 (0.81 - 0.97) p=0.01**	**0.76 (0.70 - 0.84) p<.001**	**1.14 (1.04 - 1.25) p=0.004**	0.92 (0.84 - 1.01) p=0.09	0.98 (0.89 - 1.08) p=0.70	**0.90 (0.81 – 1.00) p=0.044**
Benzodiazepines	**1.48 (1.34 - 1.62) p<.001**	**1.22 (1.10 - 1.35)[†] p<.001**	**1.96 (1.78 - 2.15) p<.001**	**1.50 (1.36 - 1.65) p<.001**	**1.45 (1.30 - 1.60) p<.001**	**1.43 (1.28 - 1.59) p<.001**

B

**Antipsychotics**	**Low dose or occasional users**		**Moderate doses**		**High doses**	
	Crude HR (95% CI)	Adjusted HR (95% CI)	Crude HR (95% CI)	Adjusted HR (95% CI)	Crude HR (95% CI)	Adjusted HR (95% CI)
Time-fixed (Cox)	1.04 (0.86 - 1.26) p=0.66	0.86 (0.70 - 1.04) p=0.11	0.97 (0.82 - 1.16) p=0.76	0.84 (0.70 - 1.01) p=0.06	**1.26 (1.06 - 1.50) p=0.01**	1.03 (0.85 - 1.24) p=0.77
Time-dependent (Cox)	**1.37 (1.13 - 1.66) p=0.001**	1.06 (0.88 - 1.29) p=0.53	**1.26 (1.06 - 1.50) p=0.01**	1.03 (0.85 - 1.23) p=0.78	**1.66 (1.39 - 1.98) p<.001**	**1.28 (1.07 - 1.55) p=0.008**
Time-dependent (NCC)[‡]	1.17 (0.95 - 1.43) p=0.15	1.06 (0.86 - 1.32) p=0.56	1.19 (0.98 - 1.44) p=0.08	1.06 (0.86 - 1.29) p=0.59	**1.47 (1.21 - 1.78) p<0.001**	**1.27 (1.03 - 1.56) p=0.02**

C

**Antidepressants**	**Low dose or occasional users**		**Moderate doses**		**High doses**	
	Crude HR (95% CI)	Adjusted HR (95% CI)	Crude HR (95% CI)	Adjusted HR (95% CI)	Crude HR (95% CI)	Adjusted HR (95% CI)
Time-fixed (Cox)	**0.72 (0.64 - 0.82) p<.001**	**0.70 (0.62 - 0.80) p<.001**	0.97 (0.86 - 1.10) p=0.64	**0.81 (0.72 - 0.92) p=0.001**	1.06 (0.92 - 1.23) p=0.3931	**0.80 (0.69 - 0.93) p=0.004**
Time-dependent (Cox)	**1.11 (0.98 - 1.27) p=0.10**	1.01 (0.89 - 1.15) p=0.87	**1.13 (1.00 - 1.28) p=0.043**	0.91 (0.80 – 1.03) p=0.14	**1.20 (1.04 - 1.39) p=0.01**	**0.86 (0.74 - 1.00) p=0.046**
Time-dependent (NCC)	1.06 (0.92 - 1.23) p=0.44	0.97 (0.84 - 1.13) p=0.73	0.94 (0.82 - 1.08) p=0.40	0.90 (0.78 – 1.03) p=0.12	0.98 (0.84 - 1.14) p=0.80	0.87 (0.74 - 1.02) p=0.10

[*] Adjusted for age, sex, social welfare with SRE and its interaction term with time, personality disorder, substance use disorder, comorbidity index and its interaction term with time, previous use of mood stabilisers, history of hospitalisation for psychosis, for another mental disorder, for non-mental reasons and its interaction term with time, number of ambulatory visits.

[†] PH assumption not respected.

[‡] For the nested-case control study, crude HR are partially adjusted since cases were matched to controls according to sex, age, comorbidity index, and prior physical hospitalisation during baseline.
Bold characters indicate statistically significant HR.

© 2024 Brodeur S et al. *JAMA Network Open.*

D

Benzodiazepines	Low dose or occasional users		Moderate doses		High doses	
	Crude HR (95% CI)	Adjusted HR (95% CI)	Crude HR (95% CI)	Adjusted HR (95% CI)	Crude HR (95% CI)	Adjusted HR (95% CI)
Time-fixed† (Cox)	**1.22 (1.10 - 1.36) p<0.001**	1.08 (0.97 - 1.20) p=0.18	**2.03 (1.79 - 2.30) p<.001**	**1.41 (1.23 - 1.60) p<.001**	**2.58 (2.11 - 3.16) p<.001**	**1.62 (1.31 - 1.99) p<.001**
Time-dependent (Cox)	**1.73 (1.56 - 1.92) p<0.001**	**1.41 (1.27 - 1.58) p<0.001**	**2.37 (2.09 - 2.68) p<0.001**	**1.57 (1.38 - 1.78) p<0.001**	**2.55 (2.09 - 3.12) p<0.001**	**1.54 (1.25 - 1.90) p<0.001**
Time-dependent (NCC)	**1.44 (1.28 - 1.62) p<0.001**	**1.42 (1.26 - 1.60) p<0.001**	**1.58 (1.38 - 1.82) p<0.001**	**1.55 (1.34 - 1.79) p<0.001**	**1.62 (1.34 – 1.98) p<0.001**	**1.54 (1.26 - 1.89) p<0.001**

© 2024 Brodeur S et al. *JAMA Network Open.*

eTable 6. Adjusted hazard ratios for all-cause mortality according to time-fixed vs. time-dependent exposure to levels of antipsychotics, antidepressants, and benzodiazepines in the more severe sub-cohort[a] (n = 18,584)

A

	**Any exposure during follow-up (5 years for survivors; until death if deceased)**		
	**Time-fixed exposure (Cox)**	**Time-dependent exposure (Cox)**	**Time-dependent exposure (NCC)**
	Adjusted HR (95% CI)	Adjusted HR (95% CI)	Adjusted HR (95% CI)
Antipsychotics	**0.73 (0.55 - 0.96) p=0.02**[b]	1.14 (0.86 - 1.50) p=0.36[†]	1.17 (0.87 - 1.58) p=0.29
Antidepressants	**0.74 (0.65 - 0.84) p<.001**	0.90 (0.79 - 1.02) p=0.11	0.93 (0.80 - 1.07) p=0.29
Benzodiazepines	**1.23 (1.08 - 1.41) p=0.003**[*]	**1.54 (1.35 - 1.76) p<.001**	**1.38 (1.20 - 1.60) p<.001**

B

**Antipsychotics**	**Low dose or occasional users**	**Moderate doses**	**High doses**
	Adjusted HR (95% CI)	Adjusted HR (95% CI)	Adjusted HR (95% CI)
Time-fixed (Cox)[†]	**0.72 (0.52 - 0.98) p=0.03**	**0.68 (0.51 - 0.90) p=0.008**	0.80 (0.60 - 1.07) p=0.13
Time-dependent (Cox)[†]	0.90 (0.66 - 1.23) p=0.51	0.86 (0.64 - 1.14) p=0.29	1.03 (0.77 - 1.37) p=0.86
Time-dependent (NCC)[c]	1.13 (0.80 - 1.59) p=0.48	1.00 (0.73 - 1.37) p=0.99	1.30 (0.95 - 1.78) p=0.098

C

**Antidepressants**	**Low dose or occasional users**	**Moderate doses**	**High doses**
	Adjusted HR (95% CI)	Adjusted HR (95% CI)	Adjusted HR (95% CI)
Time-fixed (Cox)	**0.64 (0.53 - 0.76) p<.001**	**0.80 (0.67 - 0.95) p=0.01**	0.81 (0.66 - 1.01) p=0.06
Time-dependent (Cox)	0.93 (0.78 - 1.12) p=0.46	0.89 (0.75 - 1.05) p=0.17	0.87 (0.70 - 1.08) p=0.22
Time-dependent (NCC)	1.04 (0.84 - 1.28) p=0.71	0.84 (0.69 - 1.02) p=0.07	1.02 (0.81 - 1.28) p=0.88

D

**Benzodiazepines**	**Low dose or occasional users**	**Moderate doses**	**High doses**
	Adjusted HR (95% CI)	Adjusted HR (95% CI)	Adjusted HR (95% CI)
Time-fixed (Cox)	1.10 (0.95 - 1.28) p=0.19	**1.42 (1.18 - 1.70) p<0.001**	**1.70 (1.27 – 2.28) p<0.001**
Time-dependent (Cox)	**1.47 (1.27 - 1.70) p<.001**	**1.60 (1.34 - 1.92) p<.001**	**1.58 (1.18 - 2.12) p=0.002**
Time-dependent (NCC)	**1.28 (1.08 - 1.51) p=0.004**	**1.65 (1.35 - 2.01) p<.001**	**1.92 (1.44 – 2.57) p<.001**

Abbreviations: NCC: nested-case control; HR: hazard ratio; CI: confidence interval.

[a] More severe sub-cohort: Initial cohort with at least one hospitalisation for psychosis between 2002 and 2012 inclusive

[b] Non-proportional hazards

[c] For the nested-case control study, crude HR are partially adjusted since cases were matched to controls according to sex, age, comorbidity index, and prior physical hospitalisation during the baseline period.

© 2024 Brodeur S et al. *JAMA Network Open.*

eTable 7. Comparative results of the risk estimates for all-cause mortality between studies and methods: time-fixed exposure (not corrected for ITB) and time-dependent exposure (corrected for ITB, Cox and NCC) for antipsychotics, antidepressants, and benzodiazepines (more severe sub-cohort*, n=18,584)

		Time-fixed exposure (not corrected for ITB)				Time-dependent exposure (corrected for ITB)	
	Exposure	Tiihonen, 2016[7]	Lin, 2023[8]	Li, 2024[9]	Current study	Current study (Cox)	Current study (NCC)
Antipsychotics	Low	↓S	↓S	↓S	↓S	NS	NS
	Med	↓S	↓S	↓S	↓S	NS	NS
	High	↓S	↓S	NS	NS	NS	NS
Antidepressants	Low	↓S	↓S	↓S	↓S	NS	NS
	Med	↓S	↓S	↓S	↓S	NS	NS
	High	↓S	NS	NS	NS	NS	NS
Benzodiazepines	Low	NS	↑S	↓S	NS	↑S	↑S
	Med	↑S	↑S	NS	↑S	↑S	↑S
	High	↑S	↑S	NS	↑S	↑S	↑S

Abbreviations: NCC: nested case controlled; S, statistically significant ($p < 0.05$); NS, not statistically significant ($p \geq 0.05$)

Reduced risk ↓	Increased risk ↑	
<10%	<20%	NS
10-20%	20-50%	
20-30%	50-70%	
>30%	>70%	

* More severe sub-cohort: Initial cohort with at least one hospitalisation for psychosis between 2002 and 2012 inclusive

© 2024 Brodeur S et al. *JAMA Network Open.*

eFigure 1. Selection of the study cohort (n=32,240)

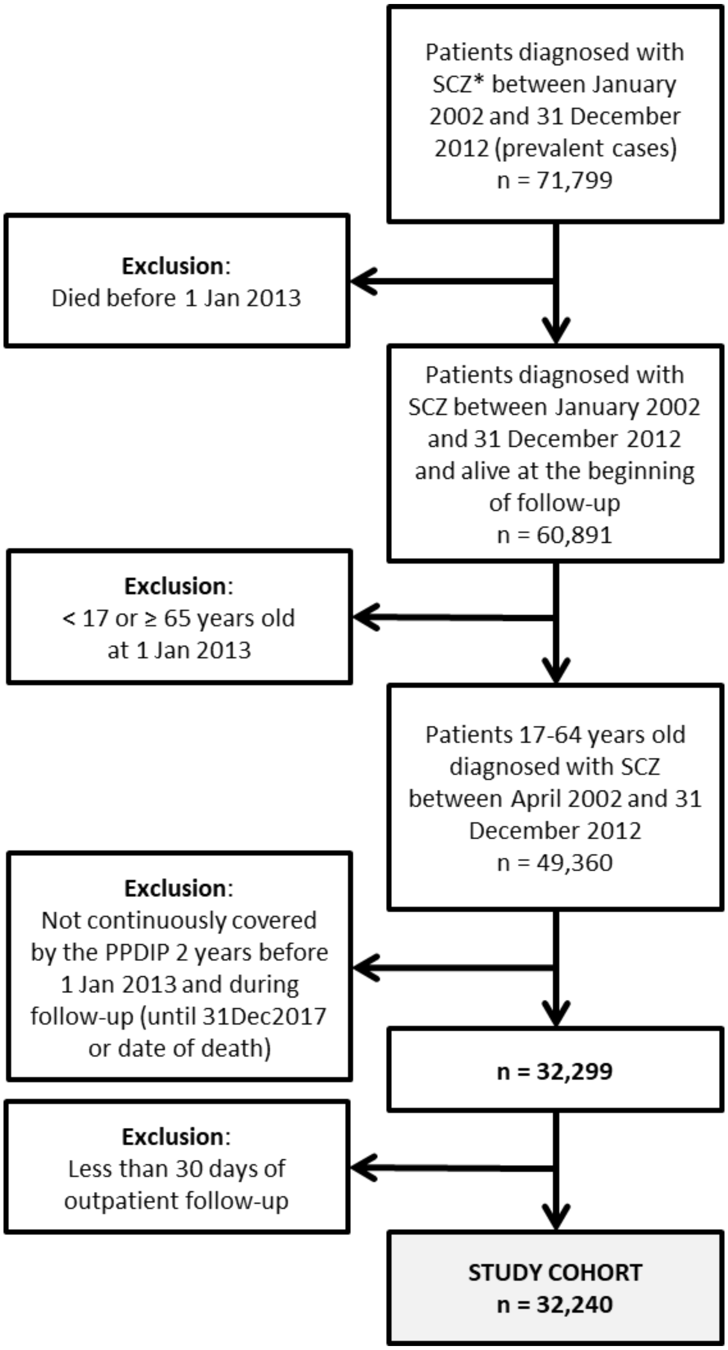

*Case definition: ≥1 hospitalisation with a principal or secondary diagnosis of SCZ or ≥2 medical services within two years (≥30 days of gap between each claim) with a diagnosis of SCZ.
Figure legend: SCZ: schizophrenia; PPDIP: public prescription drug insurance plan.

© 2024 Brodeur S et al. *JAMA Network Open.*

eFigure 2. Design of the study cohort (n=32,240)

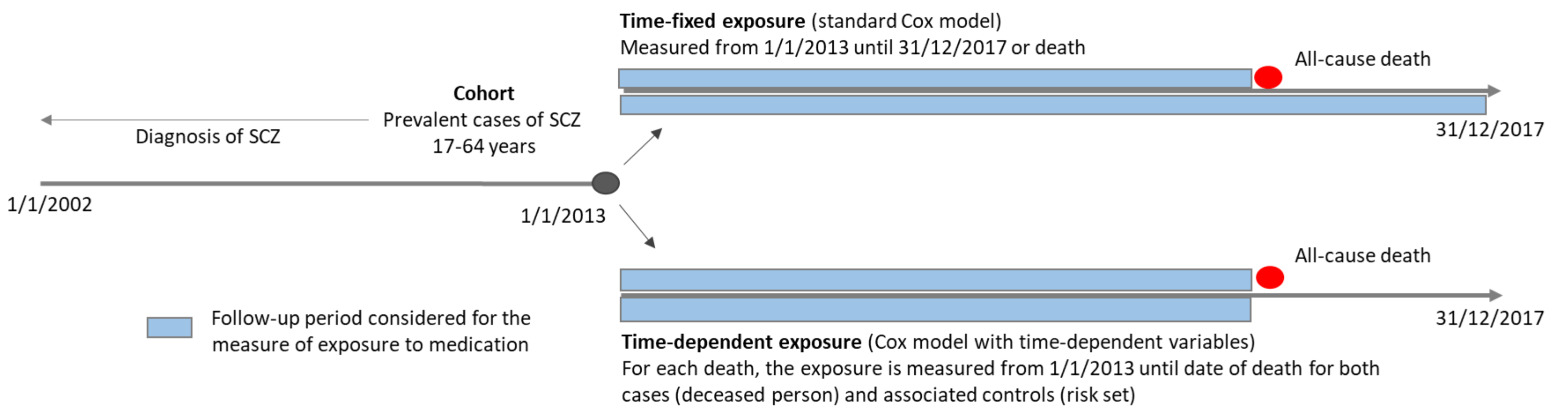

© 2024 Brodeur S et al. *JAMA Network Open.*

eFigure 3. Distribution of the study cohort according to any use of antipsychotics, antidepressants, or benzodiazepines during follow-up*

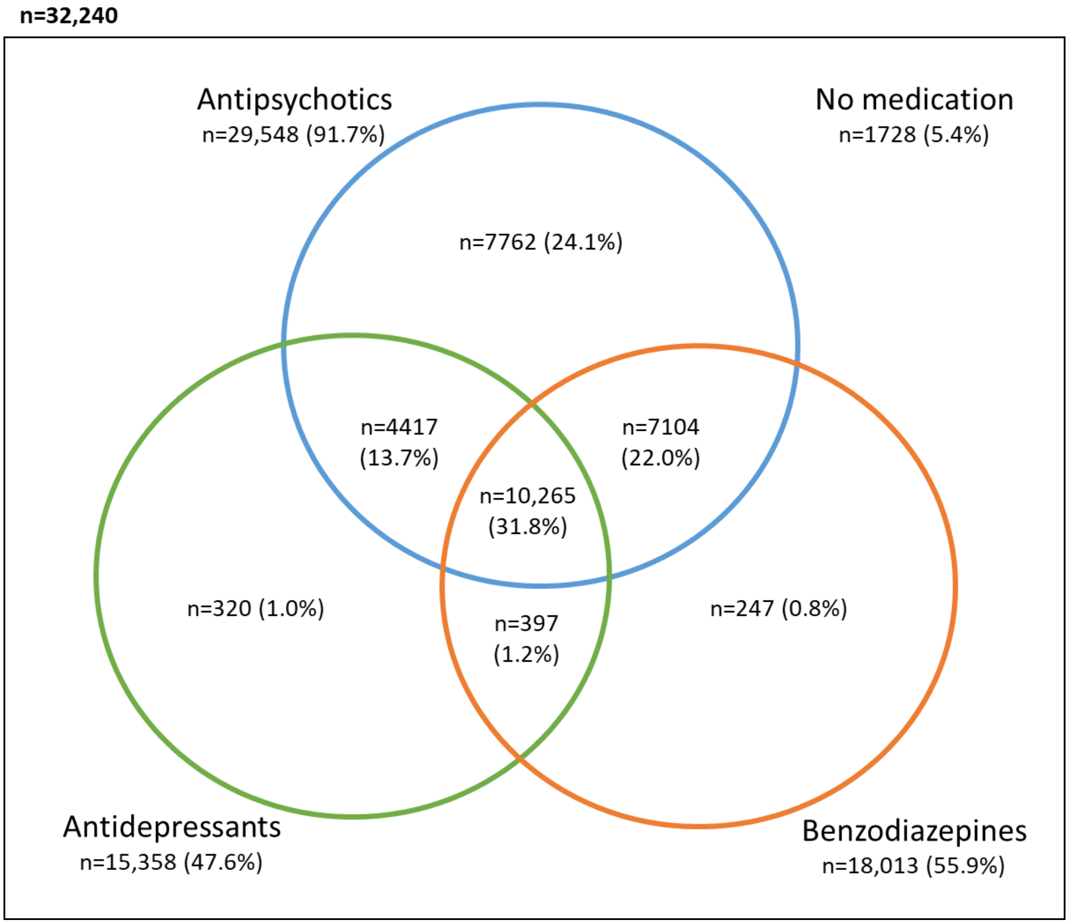

* Circle sizes are not proportional to the number of users and percentages are relative to the total

© 2024 Brodeur S et al. *JAMA Network Open.*

eFigure 4. Adjusted hazard ratios (HR) for all-cause mortality for time-fixed (not corrected for ITB) and time-dependent exposure (corrected for ITB, Cox and NCC) for binary exposure to antipsychotics, antidepressants, and benzodiazepines (n=32,240)

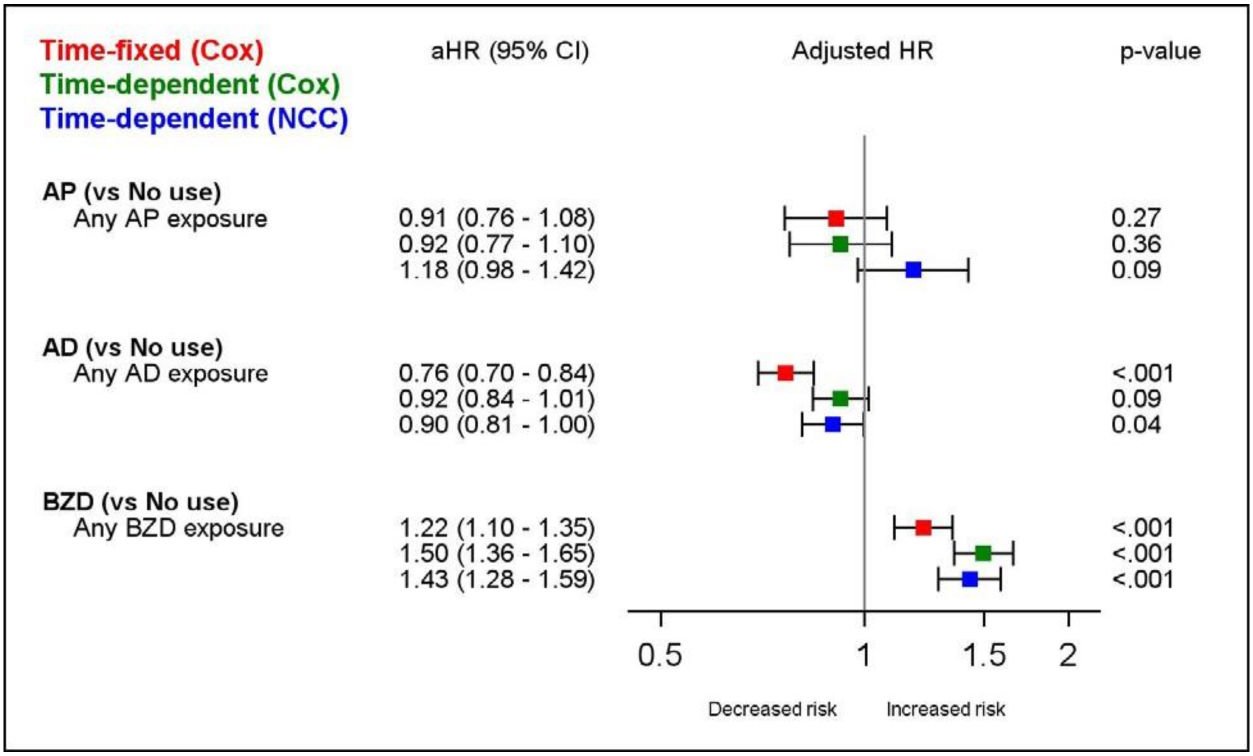

Abbreviations: AP: antipsychotics; AD: antidepressants; BZD: benzodiazepines; CI: confidence interval; aHR: adjusted hazard ratio; NCC: nested case controlled

© 2024 Brodeur S et al. *JAMA Network Open.*

eFigure 5. Adjusted hazard ratios (HR) for all-cause mortality for time-fixed (not corrected for ITB) and time-dependent exposure (corrected for ITB, Cox and NCC) for binary exposure to antipsychotics, antidepressants, and benzodiazepines (more severe sub-cohort*, n=18,584)

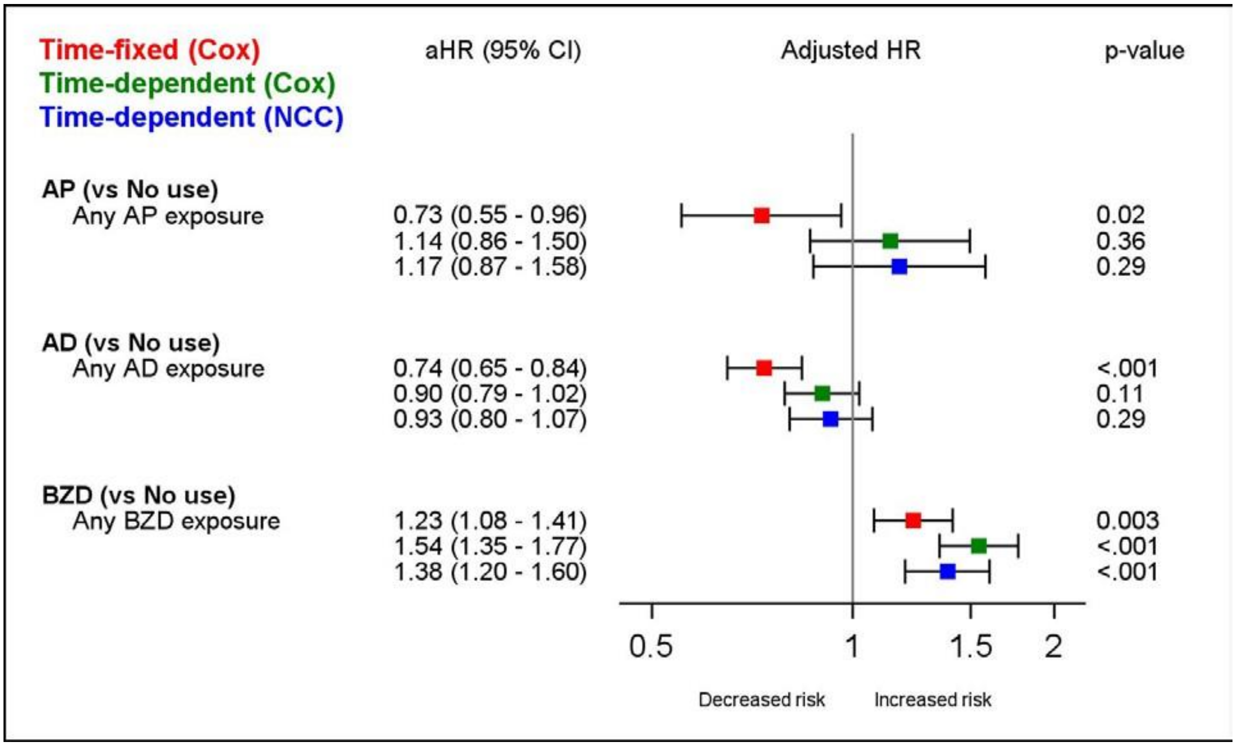

Abbreviations: AP: antipsychotics; AD: antidepressants; BZD: benzodiazepines; CI: confidence interval; aHR: adjusted hazard ratio; NCC: nested case controlled

* More severe sub-cohort: Initial cohort with at least one hospitalisation for psychosis between 2002 and 2012 inclusive

© 2024 Brodeur S et al. *JAMA Network Open.*

eFigure 6. Adjusted hazard ratios (HR) for all-cause mortality for time-fixed (not corrected for ITB) and time-dependent exposure (corrected for ITB, Cox and NCC) for antipsychotics, antidepressants, and benzodiazepines (more severe sub-cohort*, n=18,584)

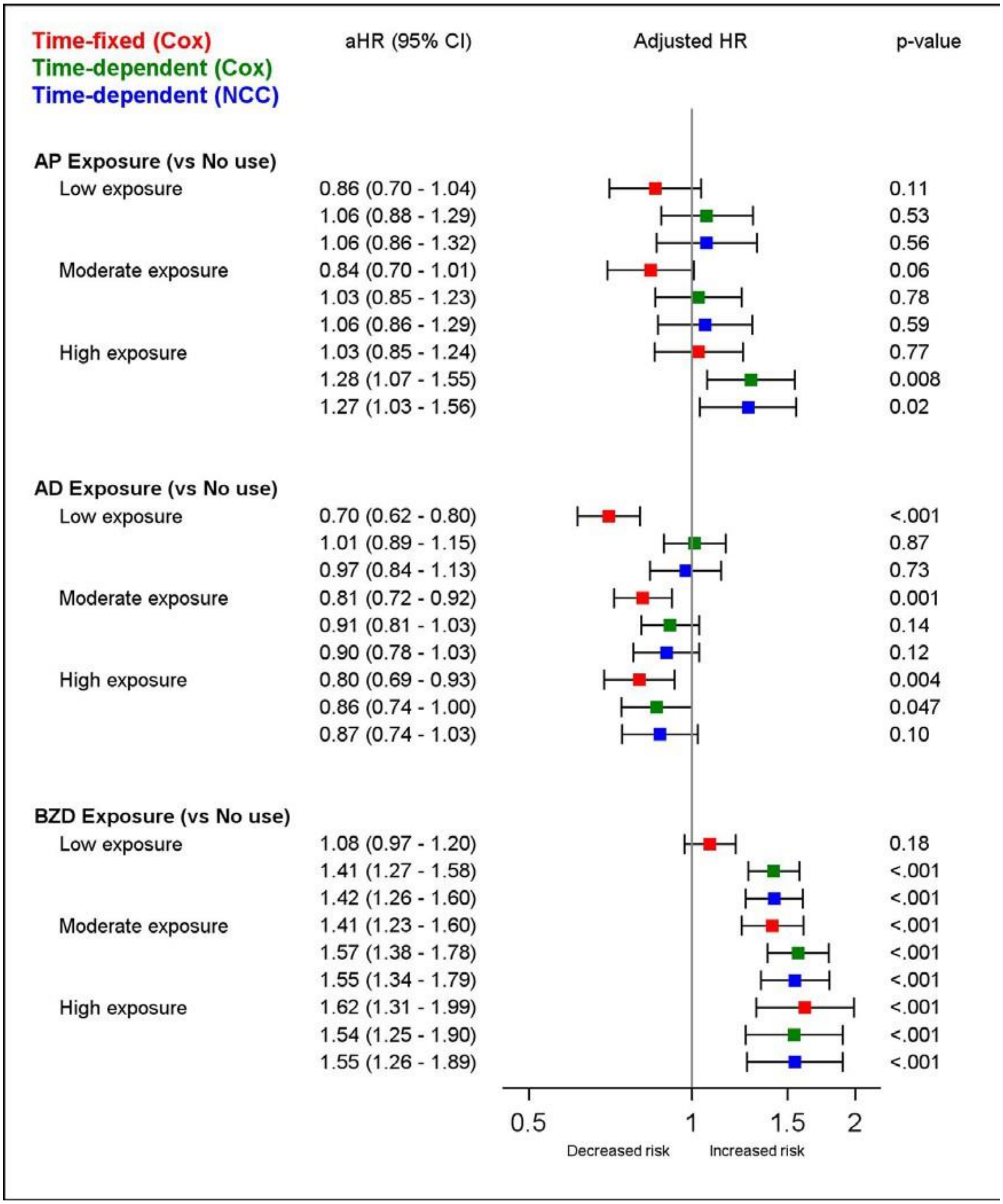

Abbreviations: AP: antipsychotics; AD: antidepressants; BZD: benzodiazepines; CI: confidence interval; aHR: adjusted hazard ratio; NCC: nested case controlled

* More severe sub-cohort: Initial cohort with at least one hospitalisation for psychosis between 2002 and 2012 inclusive

© 2024 Brodeur S et al. *JAMA Network Open.*

eFigure 7. Comparison of adjusted hazard ratios (HR) for all-cause mortality of Tiihonen et al. (2016, n=21,492, not corrected for ITB)[7], Lin et al. (2023, n=102,964, not corrected for ITB)[8], Li et al. (2024, n=6433, not corrected for ITB)[9], and the current study (more severe sub-cohort[*], n=18,584, time-fixed method, not corrected for ITB)

Abbreviations: AP: antipsychotics; AD: antidepressants; BZD: benzodiazepines; CI: confidence interval; aHR: adjusted hazard ratio; NCC: nested case controlled

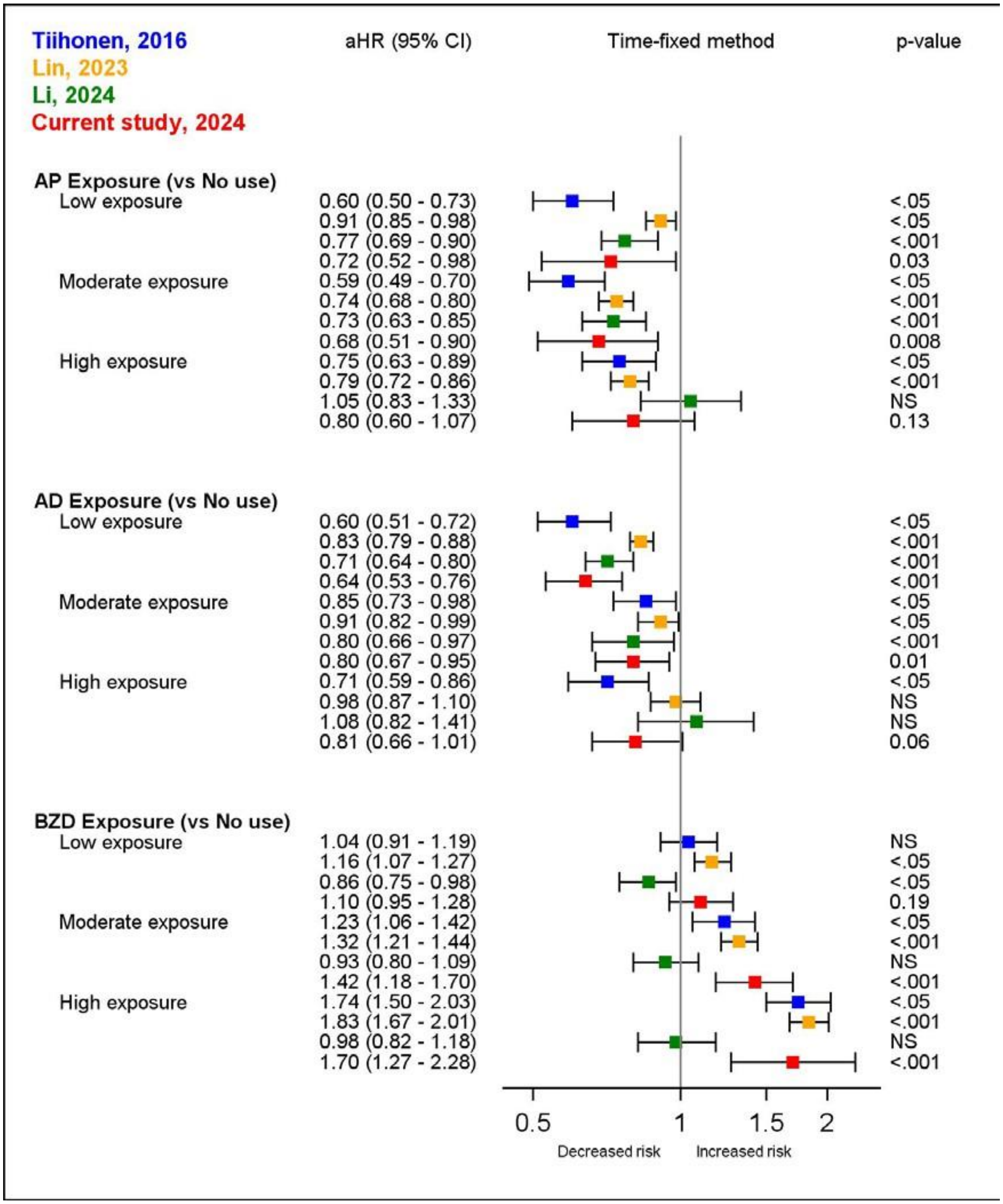

[*] More severe sub-cohort: Initial cohort with at least one hospitalisation for psychosis between 2002 and 2012 inclusive

© 2024 Brodeur S et al. *JAMA Network Open.*
